# Myxedema Coma-associated Pancytopenia: A Case Report

**DOI:** 10.5041/RMMJ.10535

**Published:** 2024-10-28

**Authors:** David A. Stein, Orit Twito, Yoav Geva, Nadav Sarid

**Affiliations:** 1 Faculty of Medicine, Tel Aviv University, Tel Aviv, Israel; 2 Department of Endocrinology, The Edith Wolfson Medical Center, Holon, Israel, Affiliated to the Faculty of Medicine, Tel Aviv University, Tel Aviv, Israel; 3 Department of Internal Medicine A, The Edith Wolfson Medical Center, Holon, Israel, Affiliated to the Faculty of Medicine, Tel Aviv University, Tel Aviv, Israel; 4 Department of Hematology, The Edith Wolfson Medical Center, Holon, Israel, Affiliated to the Faculty of Medicine, Tel Aviv University, Tel Aviv, Israel

**Keywords:** Coma, hypothyroidism, myxedema, pancytopenia

## Abstract

Pancytopenia is defined as a reduction in red blood cells, white blood cells, and platelets, and can pose as a diagnostic challenge due to the multitude of causes. Myxedema coma is the manifestation of severe untreated hypothyroidism. This case report presents a rare instance of myxedema coma-associated pancytopenia in a 53-year-old man with a history of untreated hypothyroidism. The patient presented with altered mental status and vital instability, and on further workup was found to have pancytopenia. During his hospital stay his symptomatic hypothyroidism was identified, and he was treated with intravenous levothyroxine, hydrocortisone, and supportive care. The patient’s clinical status improved gradually, with normalized blood counts upon discharge. This case underscores the significance of considering myxedema coma in the differential diagnosis of pancytopenia, especially in older patients with limited healthcare access. Increased awareness of this association can aid clinicians in timely diagnosis and management, preventing potential complications associated with untreated hypothyroidism.

## INTRODUCTION

Hypothyroidism is an extremely common disease affecting 5%–10% of the population.^1^ There are multiple causes of hypothyroidism, but the most common cause depends on geographic location and iodine access. In iodine-sufficient countries, the most common cause of hypothyroidism is autoimmune Hashimoto’s thyroiditis, while iodine deficiency is the most common cause globally.^1^ Myxedema coma is a rare but severe manifestation of untreated hypothyroidism, with an incidence of 0.22 per million per year and mortality approaching 25%. It is more commonly seen in women and the elderly.^2^ It is a feared complication characterized by symptoms of hypothyroidism, accompanied by altered mental status and potential manifestations including psychosis, hypothermia, hypotension, and bradypnea as well as disturbances in electrolyte levels like hypoglycemia and hyponatremia.^3^ Pancytopenia, the decrease in all peripheral blood lineages, is frequently encountered in routine practice. However, diagnosing pancytopenia can be challenging due to its myriad etiological factors, which include infections, nutritional deficiencies, drug side effects, as well as hematologic malignancies.^4^ Hypothyroidism, and its severe complication of myxedema coma, is considered an extremely rare cause of pancytopenia.^5^ The pathophysiology of how thyroid hormones impact hematopoiesis of all three cell lines is not well differentiated, and the existing literature on hypothyroidism-induced pancytopenia mostly consists of case reports. In the limited case reports of myxedema coma-associated pancytopenia, blood levels return after treatment with thyroid hormone, although overall prognosis did not always improve.^6^–^8^ In this case report, we describe a patient suffering from myxedema coma-associated pancytopenia whose blood counts improved after treatment.

## CASE REPORT

A 53-year-old man was admitted to the internal medicine ward with altered mental status and unstable vital signs. He was hypothermic with a rectal temperature of 33.1°C, and borderline bradycardic to 62 beats per minute. He was hypotensive with a blood pressure of 89/52 mmHg, which responded to fluid resuscitation and did not require vasopressors. His appearance was pale and generally deteriorated, and his speech was incomprehensible. There were no findings of pericardial or pleural effusions or ascites.

At admission a family member advised that the patient’s mother—the patient’s primary caretaker—had passed away a few months prior to his current hospitalization. After her passing, the patient was no longer able to care for himself, became malnourished, and stopped taking his levothyroxine medication. In this setting, he paradoxically lost over 30 kg of weight. A few days prior to admission, he started becoming more confused. The patient was found unconscious in his apartment and brought to the hospital by emergency services. He had a past medical history of congenital hypothyroidism treated with levothyroxine.

Laboratory tests at admission revealed pancytopenia (Table 1) and hyponatremia. The peripheral blood smear was unremarkable, and cells were described as normocytic and normochromic, with no signs of macrocytosis or hypersegmentation reported. No schistocytes were present. Lactate dehydrogenase (LDH) and bilirubin levels were normal. Coagulation tests (prothrombin time and fibrinogen) were normal. The patient’s cortisol was within normal limits but could have been expected to be elevated in the acute illness setting. However, it remained within normal limits after one week, so no further workup for adrenal insufficiency was deemed medically necessary at the time. Ferritin levels were elevated, but the diagnostic criteria for hemophagocytic lymphohistiocytosis were not fulfilled. Specifically, he had normal triglyceride levels (134 mg/dL; reference range 30–194), normal fibrinogen levels (271 mg/dL; reference range 200–400), and his spleen was not enlarged on computed tomography (CT). Although his ferritin level was indeed high, significantly higher levels are often seen in hemophagocytic lymphohistiocytosis. Both a COVID-19 test and urine toxicology were negative. The patient’s admission CRP was 5.8 mg/dL (reference range 0–0.5 mg/dL) and went down to 2.7 mg/dL 72 hours after admission. Neither erythrocyte sedimentation rate nor procalcitonin were measured. Chest X-ray and chest CT were unremarkable and showed no signs of an infectious process or malignancy in the thorax or upper abdomen. Head CT showed no signs of an acute intracranial pathology, vascular or infectious. The patient had no neck stiffness, and negative Kerning’s and Brudzinski signs. In light of the patient’s clinical picture, there were no indications for performing a lumbar puncture. An autoimmune panel was not performed since it was not clinically indicated at that time. Bone marrow biopsy was deferred and ultimately not performed since it was not clinically necessary in confirming the patient’s diagnosis.

**Table 1:** Laboratory Parameters of the Patient at Admission and Throughout the First Two Weeks of Hospitalization.

Parameter (reference range)	Admission	Day 1	Day 3	Day 7	Day 11	Day 19
WBC (4–10×10^3^/μL)	4.3	2.0	1.9	3.0	3.6	5.9
ANC (1.5–7.5×10^3^/UL)	3.7	1.7	1.5	1.9	2.6	5.3
Hgb (males: 13.8–17.2 g/dL)*	11.8	8.8	8.0	7.5	9.6	10.3
HCT (38%–52%)	34	25.6	23.3	22.2	28.1	30.1
MCV (80–100 fl)	79	81	81	81	84	86
PLT (150–450×10^4^/μL)	23	25	10	17	61	246
Reticulocytes (0.42%–2.23%)	-	-	0.18	-	2.18	-
Sodium (136–145 mmol/L)	127	136	141	142	141	138
TSH (0.27–4.2 μIU/mL)	71	-	-	-	29	-
Free T4 (1.0–1.6 ng/dL)	0.23	-	-	-	1.56	-
Free T3 (2.6–4.4 pg/mL)	0.96	-	-	-	-	-
Cortisol (6.2–19.4 μg/dL)	10.5	-	-	10.5	-	-
Folic acid (3.7–16.0 ng/mL)	1.8	-	-	-	-	-
Vitamin B12 (210–950 pg/mL)	1024	-	-	-	-	-
Iron (60–160 μg/dL)	190	-	-	-	-	-
Ferritin (30–400 pg/mL)	2385	-	-	-	-	-

* The patient came in malnourished and significantly dehydrated, and his blood levels on admission were therefore falsely elevated due to hemoconcentration. It was only further on during his hospitalization, and after significant volume resuscitation, that the severity of his pancytopenia was observed.

ANC, absolute neutrophil count; HCT, hematocrit; Hgb, hemoglobin; MCV, mean corpuscular volume; PLT, platelets; T3, triiodothyronine; T4, thyroxine; TSH, thyroid-stimulating hormone; WBC, white blood cell count.

The patient was stabilized and treated in an open intensive care unit. After a multidisciplinary consultation with the endocrinology and hematology/ oncology team the patient was diagnosed with myxedema coma due to untreated hypothyroidism as the most likely cause of his pancytopenia. The patient received intravenous (IV) levothyroxine 200 μg followed by oral levothyroxine 200 μg once a day, IV hydrocortisone 100 mg twice a day, and oral folic acid 5 mg once a day. The patient was initially treated with empiric antibiotic therapy for possible sepsis; however, blood cultures remained negative for bacteremia. Urine cultures returned methicillin-resistant *Staphylococcus aureus*, and treatment was de-escalated to IV vancomycin monotherapy. Within days the patient’s vital signs returned to normal. Blood counts recovered gradually (Table 1), and the patient was eventually discharged from the hospital.

## DISCUSSION

We have reported the case of a 53-year-old male who presented with altered mental status and pancytopenia and received a final diagnosis of myxedema coma-induced pancytopenia. The high index of suspicion for myxedema coma-induced pancytopenia was paramount in the apt clinical diagnosis and treatment of our patient. In a large population-based cohort study, Wouters et al. reported a prevalence of subclinical hypothyroidism of 9.4% and undetected overt hypothyroidism of 0.7%.^9^ In iodine-sufficient countries, hypothyroidism prevalence ranges from 1% to 2% in the general population and rises to 7% in those over 85.^10^ Therefore, it is not uncommon for patients to present with severe symptoms of unknown hypothyroidism. The role of thyroid hormones in erythropoiesis has been known for more than a century.^11^ Thyroid hormone significantly increases the number of burst-forming unit*-*erythroid and colony-forming unit-erythroid colonies. According to Gao et al.,^12^ thyroid hormones play a key role in terminal human erythropoiesis by acting on the thyroid hormone receptor beta. However, the mechanisms of leukopenia and thrombocytopenia caused by severe hypothyroidism are not completely understood. Nevertheless, a causal connection between thyroid hormones and bone marrow function appears likely, given that the resolution of hematologic abnormalities with thyroid replacement have also been demonstrated in the literature.^13^ Interestingly, many published cases of hypothyroidism-associated pancytopenia did not present with myxedema coma but only with profound hypothyroidism. This could be because hypothyroidism severe enough to cause pancytopenia is usually discovered before myxedema coma can develop. Furthermore, our patient had worsening pancytopenia, especially thrombocytopenia, compared to what is seen in the literature. This may point to the essential but tangential role of thyroid hormone in hematopoiesis. Yet the drastic decline in blood levels seen in our patient beyond the expected pancytopenia may have been due to general malnourishment and deterioration in his myxedema coma state. It is well established that the thyroid plays an essential role in a multitude of essential physiological processes. Furthermore, the nadir seen in our patient’s blood counts is a common phenomenon seen in other patients presenting with myxedema coma-associated pancytopenia, most likely secondary to hemoconcentration in the setting of poor oral intake.^6^ Based on our review of the literature, we calculated the average time for complete blood count recovery to be 8.5 days, with a standard deviation of 6 days. Our patient’s treatment and recovery course followed a similar pattern.

Secondary causes for our patient’s mental and physical state were considered. For differential diagnosis, infection or malignancy were considered most likely, since superimposed infections are not uncommon in myxedema coma. However, without enough objective findings during the physical exam, and in light of the laboratory results and imaging for an occult infection or malignancy, it seemed unlikely that they were the primary cause of the patient’s condition. The strongest proof of the patient’s diagnosis was his disease course and clinical picture. Our patient made a full recovery with thyroid replacement medications and stress dose steroids, without treatment for malignancy, hemophagocytic lymphohistiocytosis, or broad systemic antibiotics. In the absence of a bone marrow biopsy, this clinical course was the most indicative that the patient’s pancytopenia was due to hypothyroidism. The literature indicates that patients with pancytopenia suspected due to hypothyroidism are also given steroids, and sometimes adrenal insufficiency workups are performed.^14^,^15^ Steroid therapy or adrenal insufficiency workup should be performed in the setting of secondary hypothyroidism, or hypothyroidism of an unknown cause. On retrospective analysis of our case with congenital hypothyroidism, steroid therapy was most likely not needed.

Accordingly, routine thyroid function screening should be done as part of the investigation of new-onset pancytopenia of unknown cause.

It is important to note that patients with hypothyroidism and pancytopenia should be screened for nutritional deficiencies. Myxedema coma patients are prone to developing such deficiencies due to general deterioration and poor intake. Despite low folic acid levels, our patient did not show any classic signs of vitamin B12 or folate deficiency (macrocytosis, elevated LDH, hypersegmented neutrophils).^16^ We therefore assume that the folic acid deficiency contributed to the pancytopenia but was not the primary cause. Routine thyroid function screening should be done as part of the investigation of new-onset pancytopenia of unknown cause. Furthermore, from a retrospective perspective, our patient may have benefited from an autoimmune panel and a bone marrow biopsy. The absence of these tests represents a limitation of this case report.

## CONCLUSION

We presented the case of a 53-year-old male with a history of hypothyroidism who presented to the emergency department with myxedema coma-associated pancytopenia. After diagnostic workup and treatment of his hypothyroidism, his blood counts gradually improved over the course of 3–4 weeks. Due to the prevalence of hypothyroidism among the population, the severe manifestation of myxedema coma should be considered in the differential diagnosis of pancytopenia with an unknown cause. The role of thyroid hormone in hematopoiesis needs to be investigated further. Older patients, patients with neurological impairment, and patients with decreased access to healthcare are at a greater risk for this presentation. Basic tests like thyroid-stimulating hormone (TSH) and free thyroxine (T4) should be included in the investigation of new-onset pancytopenia of unknown cause.

## Data Availability

All data generated or analyzed during this study are included in this article.

## Abbreviations

CT: computed tomography
IV: intravenous
LDH: lactate dehydrogenase
TSH: thyroid-stimulating hormone.
